# Trend of medically induced monozygotic twin deliveries according to age, parity, and type of assisted reproductive technique during the period 2007–2017 in Lombardy Region, Northern Italy: a population-based study

**DOI:** 10.1007/s10815-021-02268-0

**Published:** 2021-07-09

**Authors:** Giovanna Esposito, Edgardo Somigliana, Matteo Franchi, Chiara Dallagiovanna, Valerio Pisaturo, Giovanni Corrao, Fabio Parazzini

**Affiliations:** 1 Department of Clinical Sciences and Community Health, University of Milan, via Augusto Vanzetti 5, 20133, Milan, Italy; 2 Department of Obstetrics, Gynecology and Neonatology, Fondazione IRCCS Ca’ Granda Ospedale Maggiore Policlinico, 20122 Milan, Italy; 3 Laboratory of Healthcare Research & Pharmacoepidemiology, Department of Statistics and Quantitative Methods, University of Milano-Bicocca, Milan, Italy; 4 National Centre for Healthcare Research and Pharmacoepidemiology, Milan, Italy

**Keywords:** Twin, Multiple pregnancy, Monozygotic, Dizygotic, ART, IVF

## Abstract

**Purpose:**

The risk of monozygotic (MZT) twinning is increased in pregnancies after assisted reproductive technologies (ART). However, determinants remain poorly understood. To shed more light on this issue, we analyzed the estimated frequency of MZT twins from ART in Lombardy, Northern Italy, during the period 2007–2017.

**Methods:**

This is a population-based study using regional healthcare databases of Lombardy Region. After having detected the total number of deliveries of sex-concordant and sex-discordant twins from ART, we calculated MZT rate using Weinberg’s method. Standardized ratios (SRs) and corresponding 95% confidence intervals (CI) of MZT deliveries, adjusted for maternal age, were computed according to calendar period, parity, and type of ART.

**Results:**

On the whole, 19,130 deliveries from ART were identified, of which 3,446 were twins. The estimated rate of MZT births among ART pregnancies was higher but decreased over time (p-value = 0.03); the SRs being 1.33 (95% CI: 1.18–1.51), 0.96 (95% CI: 0.83–1.11), and 0.92 (95% CI: 0.79–1.07) for the periods 2007–2010, 2011–2014, and 2015–2017, respectively. The SRs of MZT among women undergoing first-level techniques, conventional in vitro fertilization (IVF), and intracytoplasmic sperm injection (ICSI) were 0.47 (95% CI: 0.38–0.57), 1.02 (95% CI: 0.88–1.17), and 1.43 (95% CI: 1.27–1.61) (p-value < 0.0001). The ratio of MZT births was significantly higher in women younger than 35 years (p-value < 0.0001) and slightly higher among nulliparae (p-value < 0.0001).

**Conclusion:**

Despite a reduction of MZT rate from ART over the time, the risk remains higher among ART pregnancies rather than natural ones. Younger women and women undergoing ICSI showed the highest risk of all.

**Supplementary Information:**

The online version contains supplementary material available at 10.1007/s10815-021-02268-0.

## Introduction

Several clinical and population-based studies have consistently reported that the risk of monozygotic twin (MZT) is increased in pregnancies achieved by assisted reproductive technologies (ART) [1, 2] when compared with the natural incidence of MZT that is established to be about 0.4% of all births [3].

A recent systematic review and meta-analysis of the literature suggested that this risk is about 2.5-fold higher in ART pregnancies compared with natural conception [2]. In Lombardy, a region located in Northern Italy with more than 10 million inhabitants, we documented a 60% increased risk of MZT births among ART-treated women during the period 2010–2014 [1].

Reasons behind this association remain unclear, but it is of utmost importance to shed more light on this issue, considering that MZT pregnancies are burdened by a higher risk of adverse obstetric outcome. Two recent systematic reviews and meta-analyses tried to disentangle risk factors for MZT in pregnancies achieved by ART but failed to provide robust and consistent findings. Busnelli et al. identified the following potential risk factors: blastocyst transfer (odds ratio (OR) 2.16, 95% confidence interval (CI): 1.74–2.68), maternal age < 35 years (OR 1.90, 95% CI: 1.21–2.98), intracytoplasmic sperm injection (ICSI) (OR 1.13, 95% CI: 1.02–1.26), and assisted hatching (AH) (OR 1.17, 95% CI: 1.09–1.27) [2]. Conversely, the meta-analysis from Hviid highlighted a significant association only with the embryo transfer at blastocyst stage (OR 2.18, 95% CI: 1.93–2.48) [4]. In this regard, it has to be pointed out that the studied risk factors tend to correlate one another and meta-analyses on raw data are therefore incapable to provide firm conclusions. Fine adjusted multivariate analyses with patient individual data would be necessary to provide more definite information. However, such analyses can be carried out only in single-center studies that, however, have a low statistical power due to the rarity of the examined condition [5].

In order to provide some more information on this subject, we analyzed the frequency and trends over time of ART-related MZT deliveries using regional data routinely collected during the period 2007–2017, in Lombardy Region. In addition, we assessed the association of MZT with maternal age, parity, and different types of ART.

## Methods

We identified all deliveries that took place in Lombardy Region between 1 January 2007 and 31 December 2017 from women who had benefited from the National Health System (NHS) and resident in Lombardy. Exclusion criteria were as follows: (i) deliveries which did not match to a hospital ICD-9-CM code related to childbirth, (ii) deliveries in which the infant could not be linked to the mother, (iii) deliveries conceived spontaneously, (iv) triplets and quadruplets, (v) deliveries of mothers younger than 18 or older than 45 years at the delivery, (vi) deliveries before the 22nd week or after the 42nd week, and (vii) deliveries with a lack of information concerning the sex of at least one of the newborns.

Deliveries were identified using regional registries. Specifically, in Lombardy Region, a standard form is used to register all patients discharged from public and private hospitals (scheda di dimissione ospedaliera (SDO)). Diagnoses, interventions, and hospitalization-related costs are codified according to the International Classification of Diseases 9th edition-Clinical Modification (ICD-9-CM) and the national diagnosis-related group (DRG) system. In addition, a Certificate of Delivery Assistance is filled out at delivery (CedAP) which provides all the information on mode of conception (spontaneous/non-spontaneous (i.e., after ART or medically induced ovulation only)), maternal characteristics, pregnancy course, delivery, and obstetric and neonatal outcome at birth. A more detailed description of methodology is reported elsewhere [6].

The total number of deliveries of sex-concordant and sex-discordant twins from ART was acquired. Subsequently, dizygotic (DZT) and MZT birth rates were determined using Weinberg’s method [7]. Summarizing, it was assumed that sex gender is independently distributed in DZT, compared to MZT pregnancies. The difference between the total number of twins and twice the number of discordant twins thus provides an estimate of the number of MZT. Our study analyses were performed on the number of deliveries, not on the number of newborns.

The overall rate of twin deliveries was calculated by dividing the estimated number of twins by the total number of deliveries. This was done separately for MZT and DZT twin deliveries. Chi-squared was used for testing differences in maternal socio-demographic features according to type of pregnancy (singleton or twin). All the analyses were performed among different strata of parity and maternal age, calendar period, and type of ART (i.e., first-level techniques—pharmacological ovulation induction or intrauterine insemination (IUI), conventional in vitro fertilization (IVF), and intracytoplasmic sperm injection (ICSI)).

In order to take into account the potential confounding effect of age, adjusted rates (standardized ratios (SRs)) were calculated through a direct method of standardization. Corresponding 95% confidence interval (CI) for each rate ratio was calculated. Trends over time were investigated by dividing the study period into three intervals: 2007–2010, 2011–2014, and 2015–2017. Chi-squared was used for testing differences in SRs calculated according strata of maternal age, parity, and type of ART. In order to assess trend of twin and MZT rate over the period considered, chi-squared for trend was used.

According to Italian law, analysis of anonymous administrative database does not require Ethics Committee approval. All data were anonymous.

## Results

A total of 771,405 deliveries that occurred in Lombardy between 1 January 2007 and 31 December 2017 from women beneficiaries of National Health System (NHS) and resident in Lombardy were identified. We excluded 7,685 records because they did not match to a hospital ICD-9-CM code or to a DRG code related to childbirth, 458 records because the infant could not be linked to the mother because of a missing identification code, 742,968 records related to pregnancy conceived spontaneously, and 196 records related to multiple births (triplets or quadruplets). Finally, after exclusion of 908 deliveries because the mother was younger than 18 years or older than 45 years of age at delivery, 46 deliveries because the gestational age was too short (<22 weeks) or too long (>42 weeks), and 14 deliveries because of a lack of information concerning the sex of at least of the newborns, we obtained a final study cohort including 19,130 deliveries achieved after ART.

Among these, a total of 15,684 (82.0%) singleton and 3,446 twin (18.0%) deliveries from ART were observed.

Maternal characteristics in singleton and twin births after ART and the number of ART procedures for type (i.e., ICSI, FIVET, and first-level procedures) are shown respectively in Supplementary Table S1 and Table S2. Women who had twin pregnancies had a similar average age compared with women with singleton pregnancies (36.33 ± 4.44 vs 36.25 ± 4.37, respectively; p-value = 0.3089). Twin deliveries were significantly more common among nulliparae women (p < 0.0001), being the frequency of nulliparity 71.8% and 79.8% among singleton and twin births, respectively. No differences were observed in nationality, marital status, and education. The frequency of IVF technique increased over time, but the frequency of ICSI did not change.

Over the 10-year study period, the frequency of twin deliveries was 18.0 and 1.2/100 deliveries among ART and natural conceptions, respectively (p-value < 0.0001) (data not shown).

**Fig. 1:**
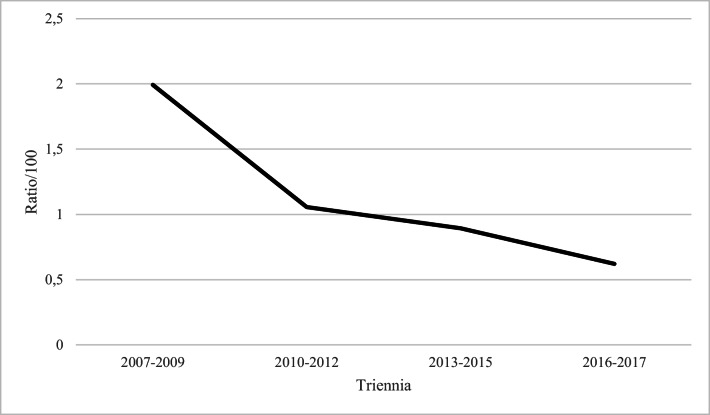
Temporal trend of estimated ratio/100 of monozygotic (MZT) delivery after assisted reproductive technologies (ART) for intervals of 3 years

On the basis of the Weinberg’s rule, the overall estimated rate of MZT deliveries after ART was 1.02/100 deliveries (95% CI: 0.89–1.17) and showed a decreasing trend; the SRs adjusted for age being 1.33 (95% CI: 1.18–1.51), 0.96 (95% CI: 0.83–1.11), and 0.92 (95% CI: 0.79–1.07) in the intervals 2007–2010, 2011–2014, and 2015–2017, respectively. The difference between the first versus the second and the third periods was statically significant (p-value = 0.03). Figure 1 shows the temporal trend of MZT after ART, observed for intervals of 3 years.

Considering the total period (2007–2017), the ratio was significantly higher among women younger than 35 years (1.51 (95% CI: 1.24–1.84)) when compared with older ones (0.78 (95% CI: 0.64–0.94)) (p-value < 0.0001). This association was consistent over the three periods, but not significant in the period 2007–2010 (p-value = 0.09) (Table 1).

**Table 1:** Monozygotic (MZT) deliveries ratio/100 according to maternal age and calendar period

Period of study	No. *	ratio/100	SR (95% CI)	p-value
2007–2010				
< 35 years	28	1.72 (1.19–2.47)	1.33 (1.18–1.51)	0.03
≥ 35 years	28	1.13 (0.78–1.62)		
2011–2014				
< 35 years	41	1.55 (1.15–2.10)	0.96 (0.83–1.11)	
≥ 35 years	34	0.66 (0.47–0.92)		
2015–2017				
< 35 years	29	1.31 (0.91–1.87)	0.92 (0.79–1.07)	
≥ 35 years	36	0.72 (0.52–0.99)		

*SR*, standardized ratio (/100)

*estimated number according to Weinberg’s method

Table 2 shows MZT frequency according to parity. We had 7,855 (41.1%) missing values regarding parity. The SR adjusted for maternal age was 0.72 (95% CI 0.61–0.85) among multiparae and 0.97 (95% CI 0.84–1.12) among nulliparae women. These differences were statistically significant (p-value < 0.0001).

**Table 2:** Monozygotic (MZT) deliveries ratio/100 according to parity in strata of maternal age

Parity	No. *	ratio/100	SR (95% CI)	p-value
Parous women				
< 35 years	13	1.58 (0.93–2.69)	0.72 (0.61–0.85)	<0.0001
≥ 35 years	6	0.27 (0.13–0.60)		
Nulliparous women				
< 35 years	47	1.61 (1.21–2.13)	0.97 (0.84–1.12)	
≥ 35 years	34	0.64 (0.46–0.89)		

*SR*, standardized ratio (/100)

*Estimated number according to Weinberg’s method

Table 3 shows MZT frequency according to type of ART. Considering the whole period, the SR adjusted for maternal age among women undergoing first-level techniques (ovarian hyperstimulation or IUI) was 0.47 (95% CI: 0.38–0.57). This was comparable with what observed among natural conceptions. Conversely, higher SRs were seen in women undergoing IVF and ICSI (1.02 (95% CI: 0.88–1.17) and 1.43 (95% CI: 1.27–1.61), respectively). The difference according to the type of ART was statistically significant (p-value < 0.0001). MZT ratio was higher among women younger than 35 years compared with older ones, both in the IVF and ICSI group. The same was not observed in the group of women undergoing first-level techniques.

**Table 3:** Monozygotic (MZT) deliveries ratio/100 according to method of assisted reproductive technologies (ART) in strata of maternal age

Method of ART	No. *	ratio/100	SR (95%CI)	p-value
First-level techniques				
< 35	7	0.47 (0.23–0.96)	0.47 (0.38–0.57)	<0.0001
≥ 35	7	0.47 (0.23–0.96)		
IVF				
< 35	38	1.98 (1.45–2.71)	1.02 (0.88–1.17)	
≥ 35	23	0.52 (0.34–0.78)		
ICSI				
< 35	48	1.89 (1.43–2.50)	1.43 (1.27–1.61)	
≥ 35	62	1.19 (0.93–1.52)		

*SR*, standardized ratio (/100)

*Estimated number according to Weinberg’s method

## Discussion

This population-based study confirmed that the risk of MZT is increased in women undergoing ART, but also highlighted a decreasing trend over time. An association between MZT and IVF or ICSI procedures emerged. Furthermore, younger women were at increased risk in statistically significant way.

As previously observed in a recent meta-analysis [2], our study showed a two-fold increase in the risk of MZT pregnancy after ART compared to natural conceptions. The overall estimated rate of MZT was 1.02/100 deliveries among women who had medically assisted pregnancies compared to about 0.40/100 deliveries among those who conceived spontaneously. A decreasing trend in the number of MZT from ART over the study period emerged. This latter aspect is conversely novel, intriguing and reassuring, although difficult to explain.

We hypothesize a role of frozen embryo transfer. This decreasing MZT trend with time could be linked to the transfer of frozen embryos, regardless their developmental stage. The progressive and widespread diffusion of frozen transfers is the most relevant change that occurred over the last 10–15 years in ART and one has therefore to first consider this potential explanation. Of utmost relevance here is the association recently described by Mateizel et al. [8] between frozen embryo transfer and lower MZT rate. The explanation for this association is difficult to provide. One may speculate a role for the endometrial environment in fresh embryo transfer. During the phase of implantation, embryos transferred in fresh cycles are exposed to a non-physiological milieu and this might cause some perturbations ultimately facilitating monozygotic twinning. If confirmed, this aspect could be an additional point in favor of a strategy of frozen transfers. This explanation would also represent a shift in our view of the problem. Indeed, up to now, research on the causes of ART-related MZT has mainly focused on laboratory procedures rather than on perturbation of the uterine environment. The observation that IUI does not increase the MZT rate despite being commonly associated to ovarian hyperstimulation does not contrast with this hypothesis. The magnitude of ovarian hyperstimulation in IUI is indeed markedly milder, actually close to physiological conditions.

Alternative possible explanations are plausible. They include subtle and elusive modifications in laboratory conditions that may have progressively occurred during the study period in the ART units of our region, such as changes in media, incubators, personnel’s expertise, and strategies for transfer. Previous studies showed an association between laboratory procedure involving micromanipulation of the zona pellucida (i.e., ICSI and AH) and MZT pregnancies [9, 10]. Moreover, blastocyst transfer was described as being associated with an increased risk of MZ twinning [8]. We may speculate that in the study period, there has been a decreasing use AH concomitant to the growing awareness that this intervention is ineffective to pregnancy rate [11]. On the other hand, an increased trend in the transfer of blastocysts versus cleavage-stage embryos has presumably occurred, and this would contrast with the observed decreased frequency of MZT, extended culture being a risk factor for MZT. Overall, one may conclude that several factors that modulate the risk of MZT have changed during the study period, of whom some increase the risk while others act in the opposite manner, with the former prevailing.

Moreover, one cannot also exclude progressive changes in the characteristics of the treated infertile population. Unfortunately, we have scant information on these aspects, but according to data from the Italian National ART Register, in Lombardy Region, the annual number of frozen embryo transfer cycles underwent a progressive growth from 102 in 2007 to 5455 in 2017 [12]. Moreover, our data highlighted an increase of IVF procedures, a steady number of ICSI procedures, and a decrease in first-level techniques markedly decreased (Table S2). Subgroup analyses aimed at highlighting the possible effects of these factors on the MZT trend could not be performed because of the insufficient sample size.

Our analysis confirmed an association between the risk of MZT and young age: women under the age of 35 showed twice as many MZT pregnancies as women over 35. To note, no population-based studies have previously showed such an association. Only clinical series were published. In particular, as highlighted in the meta-analysis by Busnelli et al., four studies evaluated the effect of oocyte age on MZT risk by estimating the incidence of this event among women aged 35 or older and younger ones [2]. The pooling of the results from the studies included in the meta-analysis showed a statistically significant increased risk among women younger than 35 years (odds ratio (OR) = 1.29, 95% CI, 1.03–1.62). Concomitantly, a second systematic review published by Hviid et al. [4] suggested a facilitating role of younger age as well, although a meta-analysis focused on this factor was not carried out.

Interestingly, in our study, this association between MZT pregnancies and young maternal age did not emerge among patients achieving natural conceptions and those undergoing first-level techniques. This observation may suggest that young age is not a direct determinant of MZT pregnancies. The role of younger age may be explained through the more common practice, among young patients, of embryo transfer at blastocyst stage, being blastocysts claimed as potential risk factor for MZT. However, this hypothesis contrasts with the observed decreasing trend of ART-related MZT pregnancies over the study period (2007–2017), given the fact that in that exact period, prolonged embryo culture and transfer at blastocyst stage has conversely increased [13]. Interestingly, using recorded time lapse imaging, Eliasen et al. found an increased percentage of MZT associated with better ICM grading [14]. They claimed as an explanation for the increased MZT in younger women the availability of better-quality embryos [14]. Previous literature was not consistent; Otsuki et al. [15] found that blastocysts containing inner cell mass classified as high grade, more common among younger rather than older patients, produced a low incidence of monochorionic diamniotic twinning. In order to justify the higher frequency of MZT among young women, the author suggested that twin embryos of older mothers were more prone to chromosomal abnormalities and miscarriage before reaching clinical pregnancy. Overall, reasons beneath the association between young age and MZT risk are still scantly understood.

Our study also highlighted a possible association between the type of ART and MZT risk, with a significant higher risk among patients undergoing conventional IVF (1.02 (95% CI: 0.88–1.17)) and the highest risk of all among women receiving ICSI (SR 1.43, 95% CI: 1.27–1.61). This evidence appeared particularly relevant in younger women. However, as previously underlined, our study design does not allow to disentangle whether these associations could be considered really causative. Noteworthy, the link between MTZ pregnancies and ICSI has been demonstrated only in the meta-analysis of Busnelli et al. [2], while Hviid et al. did not find any statistically significant association [4], nor did a recent multicenter study carried out by our group [5].

Some limitations of our study should be acknowledged. First of all, the information about the zygosity was not directly available. Thus, we could not report the real number of DZT or MZT births but we inferred them using the Weinberg’s method. This hampers the possibility of performing multivariate analyses. In this regard, it has also to be mentioned that the Weinberg’s method has not been validated for ART population yet. Secondly, information recorded on the ART procedure performed was often not precise. Detailed information about day of embryo transfer, AH, or embryonic biopsy execution is not reported in the administrative databases. Thirdly, we could not detect women who resorted to oocyte donation. This inaccuracy could be a confounder: older women transferred with embryos from donated oocytes would have to be considered equivalent to younger ones. However, the number of women undergoing oocyte donation was presumably very low in the studied period, since the procedure was forbidden in Italy until 2014 and was subsequently limited to private practice. Moreover, this bias is expected to temper the detected association between MZT and younger age rather than to overestimate it. Finally, parity data were not available for all deliveries.

Nowadays, studies available in the literature, which generally support the hypothesis of an iatrogenic role of ART in causing the increased risk of MZT, present some inconsistencies that could be due to different procedures and laboratory protocols. Too many confounders are in play, to allow us to clearly explain this association. Albeit indirect, evidence from our study is in line with this view. The decreasing trend of ART-related MZT pregnancies over time, the absence of any association with first-level techniques, and the detrimental effect of young age that emerged only in ART pregnancies but not in natural conceptions are all observations that emphasize a possible detrimental role of ART itself on MZT risk. Confounders are less likely to play a role. To date, however, the specific reasons behind this phenomenon remain obscure. Future large and multicenter studies are pressingly needed to clarify the real determinants of MZT risk in ART pregnancies. The progressive diffusion of single embryo transfer and the consequent reduction in DZT pregnancy incidence has brought out the problem of MZT pregnancies that has been hidden for years. Preventing MZT pregnancies in ART is the new challenge and warrants joined efforts of the scientific community.

## Supplementary Information

ESM 1 (DOCX 18 kb)

ESM 2 (DOCX 14 kb)

## Data Availability

The data that support the findings of this study are available from Lombardy Region, but restrictions apply to the availability of these data which were used under license for the current study, and so are not publicly available. Data are however available from the authors upon reasonable request and with permission of Lombardy Region.

## Abbreviations

AH: Assisted hatching
ART: Assisted reproductive technologies
CeDAP: Certificates of delivery assistance
CI: Confidence interval
DRG: Diagnosis-related group
DZT: Dizygotic twin
ICD-9-CM: International Classification of Diseases 9th edition-Clinical Modification
ICSI: Intracytoplasmic sperm injection
IUI: Intrauterine insemination
IVF: In vitro fertilization
MZT: Monozygotic twin
NHS: National Health System
OR: Odds ratio
SDO: Scheda di dimissione ospedaliera
SR: Standardized ratio
