# Supplementary material for: Trend of medically induced monozygotic twin deliveries according to age, parity, and type of assisted reproductive technique during the period 2007–2017 in Lombardy Region, Northern Italy: a population-based study
Source: J Assist Reprod Genet. 2021 Jul 9;38(9):2341–7. doi: 10.1007/s10815-021-02268-0 (PMC8490568; doi:10.1007/s10815-021-02268-0)
Supplement: Supplementary file 1 — (DOCX 18 kb) [file 10815_2021_2268_MOESM1_ESM.docx]

**Table S1. Maternal characteristics in the cohort of** **19,130 pregnancies obtained after assisted reproductive technologies (ART). Lombardy, Italy. 2007-2017.**

|  | **Singletons (N=15,684)** | **Twins (N=3,446)** | **p-value** |
| --- | --- | --- | --- |
| *Year of birth* | |  | |
| 2007 | 630 (4.0) | 142 (4.1) | <0.0001 |
| 2008 | 787 (5.0) | 177 (5.1) |  |
| 2009 | 822 (5.2) | 202 (5.9) |  |
| 2010 | 1,080 (6.9) | 275 (8.0) |  |
| 2011 | 1,468 (9.4) | 363 (10.5) |  |
| 2012 | 1,586 (10.1) | 340 (9.9) |  |
| 2013 | 1,536 (9.8) | 386 (11.2) |  |
| 2014 | 1,695 (10.8) | 418 (12.1) |  |
| 2015 | 1,837 (11.7) | 394 (11.4) |  |
| 2016 | 2,096 (13.4) | 397 (11.5) |  |
| 2017 | 2,147 (13.7) | 352 (10.2) |  |
| *Maternal age (years)* | |  | |
| <35 | 5,301 (33.8) | 1,188 (34.5) | 0.4479 |
| >=35 | 10,383 (66.2) | 2,258 (65.5) |  |
| *Mean* *age* | 36.25±4.37 | 36.33±4.44 | 0.3089 |
| *Parity ^a^* |  |  |  |
| Nulliparae | 6,612 (71.8) | 1,651 (79.8) | <0.0001 |
| Parae | 2,595 (28.2) | 417 (20.2) |  |
| *Nationality* | |  | |
| Italian | 14,188 (90.5) | 3,108 (90.2) | 0.6258 |
| Not Italian | 1,496 (9.5) | 338 (9.8) |  |
| *Marital status ^b^* | |  | |
| Married | 11,894 (76.9) | 2,651 (78.5) | 0.0441 |
| Not married | 3,564 (23.1) | 724 (21.5) |  |
| *Maternal education ^c^* | |  | |
| Middle school | 2,177 (13.9) | 484 (14.1) | 0.2941 |
| High school | 6,838 (43.6) | 1,546 (44.9) |  |
| University | 6,650 (42.5) | 1,412 (41.0) |  |
| *Maternal employment ^c^* | |  | |
| Employed | 13,497 (86.1) | 2,951 (85.6) | 0.5144 |
| Not employed | 2,186 (13.9) | 495 (14.4) |  |

^a^ Not including 7,855 missing data.

^b^ Not including 297 missing data.

^c^ Not including 23 missing data.

^d^ Not including 1 missing data.
