# Supplementary material for: Trend of medically induced monozygotic twin deliveries according to age, parity, and type of assisted reproductive technique during the period 2007–2017 in Lombardy Region, Northern Italy: a population-based study
Source: J Assist Reprod Genet. 2021 Jul 9;38(9):2341–7. doi: 10.1007/s10815-021-02268-0 (PMC8490568; doi:10.1007/s10815-021-02268-0)
Supplement: Supplementary file 2 — (DOCX 14 kb) [file 10815_2021_2268_MOESM2_ESM.docx]

**Table S2. Type of assisted reproductive technologies (ART) according to calendar year.**

| *Year of birth* | **First level procedures (N=2,991)** | **IVF (N=6,366)** | **ICSI (N=7,750)** |
| --- | --- | --- | --- |
| 2007 | 213 (7.1) | 208 (3.3) | 283 (3.7) |
| 2008 | 253 (8.5) | 266 (4.2) | 370 (4.8) |
| 2009 | 213 (7.1) | 280 (4.4) | 437 (5.6) |
| 2010 | 261 (8.7) | 383 (6.0) | 588 (7.6) |
| 2011 | 323 (10.8) | 548 (8.6) | 765 (9.9) |
| 2012 | 313 (10.5) | 589 (9.3) | 793 (10.2) |
| 2013 | 278 (9.3) | 652 (10.2) | 803 (10.4) |
| 2014 | 280 (9.4) | 696 (10.9) | 900 (11.6) |
| 2015 | 295 (9.9) | 806 (12.7) | 901 (11.6) |
| 2016 | 303 (10.1) | 941 (14.8) | 957 (12.3) |
| 2017 | 259 (8.7) | 997 (15.7) | 953 (12.3) |
